# Genome-wide association study on dairy goat milk production traits using three models

**DOI:** 10.3389/fgene.2025.1650836

**Published:** 2025-08-22

**Authors:** Zhengang Huang, Yuanping Tang, Jianyu Zhou, Dongliang Xu, Xiaokun Lin, Ming Cheng, Jianguang Wang, Qinan Zhao, Jianning He, Xiaoxiao Gao, Jinshan Zhao, Hegang Li

**Affiliations:** ^1^ Qingdao Agricultural University, Qingdao, China; ^2^ Qingdao Institute of Animal Husbandry and Veterinary Medicine, Qingdao, China; ^3^ Inner Mongolia Shengjian Biotechnology Co., Ltd, Hohhot, China; ^4^ Inner Mongolia Academy of Agricultural and Animal Husbandry Sciences, Hohhot, China

**Keywords:** genome-wide association study, candidate genes, milk production, dairy goat, significant SNPs

## Abstract

**Introduction:**

Identifying genetic markers associated with economically important traits in dairy goats helps enhance breeding efficiency, thereby increasing industry value. However, the potential genetic structure of key economic traits in dairy goats is still largely unknown.

**Methods:**

This study used three genome-wide association study (GWAS) models (GLM, MLM, FarmCPU) to analyze dairy goat milk production traits (milk yield, fat percentage, protein percentage, lactose percentage, ash percentage, total dry matter, and somatic cell count). The goal was to identify SNPs and positional and functional candidate genes significantly associated with these traits.

**Results:**

The GWAS analysis results identified a total of 242 significant SNPs. Among these, 45 SNPs exhibited genome-wide significance, while 197 SNPs demonstrated suggestive associations, corresponding to 99 positional candidate genes within a 50 kb upstream and downstream range. 15 significant SNP loci were consistently identified across all three models, corresponding to 18 candidate genes.The integrated analysis of three models detected 2, 19, 17, 4, 115, 23, and 62 significant SNPs associated with milk yield, ash percentage, protein percentage, lactose percentage, somatic cell count, fat percentage, and total dry matter percentage, respectively. Correspondingly, 6, 24, 9, 12, 37, 14, and 30 candidate genes were identified for these traits. Additionally, several new candidate genes related to milk production traits were proposed (LCORL, TNFRSF1A, VWF, SPATA6, MAN1C1, MASP1, BRCA2).

**Discussion:**

In summary, the results of this study provide an important reference for further exploration of the genetic mechanisms underlying dairy goat milk production traits and the development of molecular breeding markers.

## 1 Introduction

China is a major country in goat farming and livestock product production, ranking first in the world for both the number of goats and the volume of goats slaughtered (Zhang et al., 2020). It ranks fourth in the world for goat milk production (Zhang et al., 2020). According to statistics, China has nearly 5 million dairy goats, producing 1.07 million tons of goat milk, which accounts for 5.7% of the world’s goat milk production and 3.4% of the national milk production (Guo et al., 2023). In the ranking of average milk yield per dairy goat, China is 37th, with an average of 183.6 kg per goat, which is above the world average level (Luo et al., 2019). Although China has one of the largest numbers of dairy goats in the world, the average milk yield per goat is still quite low. Therefore, improving the productivity of Chinese dairy goats is crucial for the development of the dairy goat industry in China.

Goat milk, as the main economic product of dairy goats, has always been an important component of human nutrition. Compared to cow milk, goat milk is more similar to human breast milk, offering higher nutritional value, better digestibility, and greater nutritional utilization (Feng et al., 2019). However, the production of goat milk is significantly lower than that of cow milk, and research on the components of goat milk is also much less than that on cow milk. Therefore, identifying important genetic markers and candidate genes associated with milk yield and milk components in dairy goats holds significant research value.

Genome-wide association studies (GWAS) are a method for exploring the genetic architecture of important economic traits and have now become a crucial method for detecting candidate genes for complex quantitative traits in livestock and poultry (Wittenburg et al., 2020). To date, several countries have conducted GWAS on different breeds of dairy goats and achieved certain results. For example, France has conducted GWAS on the milk production traits of Saanen and Alpine dairy goats and identified candidate genes related to these traits (Martin et al., 2017; Martin et al., 2018; Massender et al., 2023; Talouarn et al., 2020), Spain (Luigi-Sierra et al., 2024), New Zealand (Scholtens et al., 2020), Canada (Massender et al., 2023), the United States (Tilahun et al., 2020), the United Kingdom (Mucha et al., 2018), and Russia (Selionova et al., 2024). Several genes have been reported to be associated with milk production traits, significantly affecting milk composition. For example, Eid et al., 2020 detected a significant mutation T713C in the DGAT1 gene in Zaraibi goats, which can significantly regulate total milk solids and milk yield. Martin et al. (2017) found that the R251L and R396W mutations impact fat content reduction. Dettori et al. (2023) discovered that the *CSN1S2* gene is not only related to milk protein and fat content but also to milk yield and other milk traits (pH value, SCS, NaCl). Massender et al. (2023), through GWAS, found that casein genes are important for milk production traits in Saanen and Alpine dairy goats.

The three models used in this study are GLM, MLM, and FarmCPU. The GLM model uses PCA as a covariate, which can reduce false positives caused solely by population structure (Price et al., 2006). The MLM model uses PCA and the K matrix to reduce false positives arising from both population structure and familial relationships (Yu et al., 2006). FarmCPU is a multi-locus model that uses a multi-locus linear mixed model (MLMM) and gradually incorporates multiple markers as covariates in the MLM to partially eliminate the confounding between the test marker and kinship (Liu et al., 2016). It has been reported that GLM and MLM are superior to ANOVA in controlling false positives, with MLM being superior to GLM (Yu et al., 2006). The MLM model can suppress the excessive inflation of p-values, thereby reducing false positives, but it also leads to an increased probability of false positives (Zhang et al., 2010). The FarmCPU model has been reported to better control the occurrence of false positives and false negatives compared to other models (Liu et al., 2016).

Therefore, the aim of this study is to use three models of genome-wide association analysis to identify SNPs and important candidate genes that are significantly associated with milk production traits.

## 2 Methods

### 2.1 Sample collection and phenotypic measurements

A total of 208 healthy lactating Guanzhong dairy goats (parity 2-4, lactation days 60–150) were randomly selected using a random number table at Shenda Ranch in Hohhot, Inner Mongolia. Jugular venipuncture was performed to collect 5 mL of EDTA-anticoagulated whole blood, which was immediately stored at −80 °C until genomic DNA extraction using the MagCore^®^ Genomic DNA Blood Kit. The phenotypic data collected from dairy goats encompassed 22-day lactation milk production records, milk composition analysis data, and somatic cell count (SCC) in milk. Milk yield was recorded daily using milking machines, milk composition was analyzed by MilkoScan FT + infrared analyzer, and somatic cell count (SCC) was determined via Fossomatic 5,000 flow cytometry. Each milk sample underwent individual analysis to ensure the accuracy of the data pertaining to 7 milk production traits. These traits are as follows: milk yield (MY), fat percentage (FP), protein percentage (PP), lactose percentage (LP), ash percentage (AP), total dry matter (TDM), and somatic cell count (SCC).

### 2.2 Genotyping and quality control

Genomic DNA was extracted from tissue samples using the MagPure Tissue DNA Kit (Thermo Fisher), with quality assessed by Qubit 4.0 fluorometry and 1% agarose gel electrophoresis (OD260/280 = 1.8–2.0). DNA fragmentation to 300–500 bp was performed using a Covaris M220 ultrasonicator, followed by end repair, A-tailing, and adapter ligation to construct Illumina-compatible libraries. Library quality was verified via Agilent 2,100 Bioanalyzer (fragment size deviation ±10%). Sequencing was conducted on the MGI-Seq 2000 platform using probe-anchor synthesis technology (PE150 mode), with raw data quality control performed by FASTP v0.23.2. Chen et al. (2018), following these detailed steps (Zhang et al., 2020): Low-quality read filtering: Reads were excluded if more than 50% of the bases had a quality score (Q ≤ 20) (Guo et al., 2023). Adapter trimming: Removal of adapter sequences from the reads (Luo et al., 2019). Excessive N base filtering: Reads containing more than 5 N bases were discarded (Feng et al., 2019). Length filtering: Reads shorter than 100 bases were filtered out. Subsequently, alignment and variant detection of the sequencing data were performed using Sentieon software version 202,308.03 (Kendig et al., 2019). Quality control of the filtered data was performed using Plink 1.9 (Chang et al., 2015), adhering to the following stringent criteria: samples with a call rate below 95% were excluded, and SNPs that did not meet quality standards were filtered out. Specifically, all genotypes had a call rate of no less than 90%, the minor allele frequency (MAF) was no less than 5%, and the Hardy-Weinberg equilibrium test for SNP genotypes with a significance level of *P* < 1 × 10^−6^ (Tuersuntuoheti et al., 2023). Additionally, the retained SNPs must also meet the conditions of an average sequencing depth ≥5× and calling quality ≥20.

### 2.3 Population stratification

Genetic distances between samples were calculated, and a phylogenetic tree was constructed using Plink 1.9 for 15,511,550 SNP sites. Principal component analysis (PCA) was also performed using Plink 1.9 to cluster the sample populations based on these sites. The population genetic structure of 208 dairy goats was analyzed using Admixture software version 1.3.0 (Kars et al., 2021).

### 2.4 Genome-wide association studies

To determine the association between SNP and milk production traits, we conducted a genome-wide association study (GWAS) using the rMVP package in R software version 4.0.4 (Yin et al., 2021). Three models were employed: the General Linear Model (GLM), the Mixed Linear Model (MLM), and FarmCPU. Milk yield and milk composition data are utilized as the phenotype, with sample age incorporated as a covariate in the models. While GLM, MLM, and FarmCPU are widely used in GWAS analysis, they each have distinct differences.

The following three models, GLM, MLM, and FarmCPU, were used respectively for genome-wide association analysis:

y=Wα+xβ+ε,

y=Wα+xβ+μ+ε,

y=Wα+S+ε=μ+ε,

Where y is the vector of phenotypes for the analyzed trait; n samples correspond to n traits; W is the fixed effects matrix; α is the corresponding coefficient including the intercept; x represents the SNP genotype; β represents the effect size of the marker; μ is the random effect; ε represents the residuals; S represents the quantitative trait SNP.

To confirm the significant impact of SNPs and identify important regions within the studied goat genome, a Bonferroni correction was performed. The *P*-value threshold of 3.22 × 10^−9^ indicates genome-wide significance, while a p-value threshold for suggestive associations of 6.45 × 10^−8^. To avoid false negatives caused by the stringent Bonferroni correction, we adjusted the *P*-value threshold for milk yield traits to 5 × 10^−9^ for genome-wide significance and 1 × 10^−7^ for suggestive associations. Manhattan plots and QQ plots were generated using R software version 4.0.4, with Manhattan plots illustrating the distribution of significant SNPs across the chromosomes, and QQ plots demonstrating the normal distribution characteristics of the data.

### 2.5 Functional analysis

Based on the gene annotation information of ARS1 in the Ensembl database (Index of/pub/release-113/gtf/capra_hircus), the GALLO package in R software version 4.2.2 is used to retrieve positional candidate genes within a 50 kb upstream and downstream range of a significant SNP. The online tools Metascape (https://metascape.org/gp/index.html) and KOBAS (https://bioinfo.org/kobas) are utilized to perform GO and KEGG enrichment analysis on the positional candidate genes to determine their biological functions and identify common pathways involved. Finally, the online tool STRING (https://cn.string-db.org) is used to construct a gene interaction network based on the predicted proteins of the positional candidate genes.

## 3 Results

### 3.1 Whole genome resequencing

The sequencing output data (clean data) for all samples totaled 6,317.42 Gb, with an average of 30.37 Gb per sample. The average Q30 value for all samples was 94.97%. The sequencing depth of 208 dairy goats was greater than 5×, with an average coverage of 90.60%. Only biallelic SNPs located on chromosomes were retained, resulting in a dataset comprising 15,511,550 SNPs for subsequent analysis.

### 3.2 Phenotypic statistics

Table 1 presents the descriptive statistics for milk yield and milk parameters of dairy goats used in this study. Analysis of the obtained data indicates that lactose percentage and total solids ratio are the parameters with the least variability, with coefficients of variation (CV) not exceeding 15%. The coefficients of variation for milk yield, milk fat percentage, milk protein percentage, and ash percentage range between 20% and 35%, which is consistent with data from other studies. Somatic cell count exhibits significant variability, with a CV exceeding 120%.

**TABLE 1:** Descriptive statistics of milk parameters in dairy goat study samples.

Trait^a^	Max	Min	Mean	SD	CV, %
MY, kg/d	3.603	0.90	2.07	0.578	27.9
FP, %	7.93	0.28	4.15	1.38	33.3
PP, %	8.86	2.52	3.81	0.90	23.6
LP, %	6.33	2.57	4.30	0.54	12.6
AP, %	1.59	0.45	0.67	0.14	20.9
TDM, %	27.03	9.90	13.13	1.95	14.9
SCC, k/mL	14,079	32	1,491.8	1925.2	129.1

^a^ MY, milk yield; AP, ash percentage; PP, protein percentage; LP, lactose percentage; SCC, somatic cell count; FP, fat percentage; TDM, total dry matter percentage.

### 3.3 Population stratification

The phylogenetic tree constructed based on quality-controlled SNP markers is shown in Supplementary Figure S1. It can be observed that there is a certain similarity in the genetic characteristics of the sample populations. Principal component analysis (PCA) of the dairy goat populations revealed that the sample populations are relatively concentrated. According to the first and second principal components, the individual differences within the dairy goat population are small (Supplementary Figure S2). To determine the appropriate number of subgroups (K), we assumed K values ranging from 1 to 9 and calculated the cross-validation error for each breed assigned to the K-th subgroup. The results showed that when K = 2, the CV error stabilized, so K = 2 was chosen as the optimal number of subgroups. The distribution of cross-validation errors at different K values is shown in Supplementary Figure S3, and the population structure is illustrated in Supplementary Figure S4.

### 3.4 Significant SNP

**FIGURE 1:**
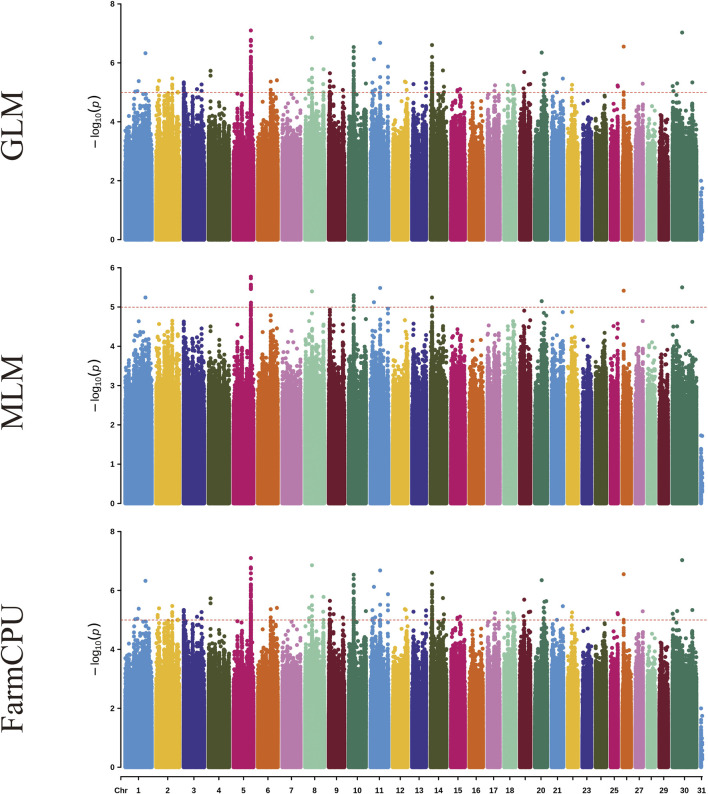
Manhattan plots for milk yield traits in dairy goats across three different models. Each plot, from top to bottom, represents the GLM, MLM, and FarmCPU models. On the Y-axis, the red line indicates the significance level of p ≤ 0.00001.

Figure 1 and Supplementary Figure S6 shows the visualization of statistically significant polymorphic positions on 30 chromosomes for certain milk traits in dairy goats.

Using genome-wide association analysis, a total of 242 significant SNPs were identified across all traits. Among these, 45 SNPs exhibit genome-wide significance, while 197 SNPs demonstrate suggestive associations. The three models identified significant SNP for milk yield, ash percentage, protein percentage, lactose percentage, somatic cell count, fat percentage, and total dry matter percentage, with 2, 19, 17, 4, 115, 23, and 62 significant SNP respectively (Table 2). These SNP are distributed across 27 chromosomes (1, 2, 3, 4, 5, 6, 7, 8, 12, 13, 14, 15, 16, 17, 18, 19, 20, 21, 22, 23, 24, 25, 26, 27, 28, 29, X).

**TABLE 2:** Number of significant SNP and annotated genes within 50 kb upstream and downstream for each trait in dairy goats across different models.

Trait^a^	Model					
	GLM		MLM		FarmCPU	
	SNP	Genes	SNP	Genes	SNP	Genes
MY	2 (0)	6	0 (0)	0	2 (0)	6
FP	18 (3)	11	4 (0)	7	7 (4)	4
PP	17 (0)	9	5 (0)	8	13 (0)	9
LP	4 (0)	12	0 (0)	0	4 (0)	12
AP	15 (6)	14	7 (3)	8	11 (8)	10
TDM	56 (12)	24	10 (3)	13	10 (5)	13
SCC	111 (24)	33	26 (2)	11	9 (5)	8

^a^ MY, milk yield; AP, ash percentage; PP, protein percentage; LP, lactose percentage; SCC, somatic cell count; FP, fat percentage; TDM, total dry matter percentage.

In the GLM model for the somatic cell count trait, the highest number of significant SNP was identified, though there may be false positives among these significant SNP. For the milk yield trait, no significant SNP were identified for genome-wide significance, and no significant SNP were identified in the MLM model. In the FarmCPU model for the ash percentage trait, the most significant SNP locus (15:59125909, *P* = 3.10 × 10^−22^) was located on chromosome 15. Additionally, the FarmCPU model identified extremely significant SNP for somatic cell count, fat percentage, and total dry matter percentage traits, located on chromosome 13 (13:2275920, *P* = 4.14 × 10^−16^), chromosome 3 (3:14938807, *P* = 5.75 × 10^−18^), and chromosome 20 (20:66155928, *P* = 1.03 × 10^−18^), respectively.

### 3.5 Positional candidate genes

To further investigate the molecular markers and functional genes related to economic traits in goat milk, we identified genes within a 50 kb range upstream and downstream of the significant SNP as positional candidate genes. Based on annotation information, we found a total of 99 positional candidate genes. Supplementary Table S1 lists the positional candidate genes identified from the significant SNP found for each trait across the three different models. In dairy goats, 6, 24, 9, 12, 37, 14, and 30 positional candidate genes were identified for milk yield, ash percentage, protein percentage, lactose percentage, somatic cell count, fat percentage, and total dry matter percentage traits, respectively. These genes are located on 20 different chromosomes (1, 2, 3, 4, 5, 6, 7, 8, 12, 13, 14, 16, 17, 18, 20, 21, 22, 25, 27, 28). As shown in Supplementary Tables S1 15 significant SNP loci were consistently identified across all three models, corresponding to 18 positional candidate genes. Additionally, 70 significant SNP loci were shared between any two models, which were associated with 35 candidate genes.

Among the identified positional candidate genes, we found that 15 genes were identified across multiple traits. The genes *CCDC154*, *CLCN7*, *GNPTG*, *PTX4*, *TELO2*, *UNKL*, and *UQCC4* were identified in traits for ash percentage, protein percentage, fat percentage, and total dry matter percentage. Other genes were identified in different traits. For instance, the genes *BLVRB*, *HIPK4*, *PRX*, *SERTAD1*, *SERTAD3*, and *SPTBN4* were identified in ash percentage and total dry matter percentage traits; *LCORL* was identified in protein percentage and total dry matter percentage traits; *SELENOF* was identified in fat percentage and total dry matter percentage traits. The same genes were identified in the four traits of ash percentage, protein percentage, fat percentage, and total dry matter percentage, while no genes were identified as common between milk yield, somatic cell count, and lactose percentage traits. All genes related to multiple traits were identified in the total dry matter percentage trait.

### 3.6 Functional analyses

**FIGURE 2:**
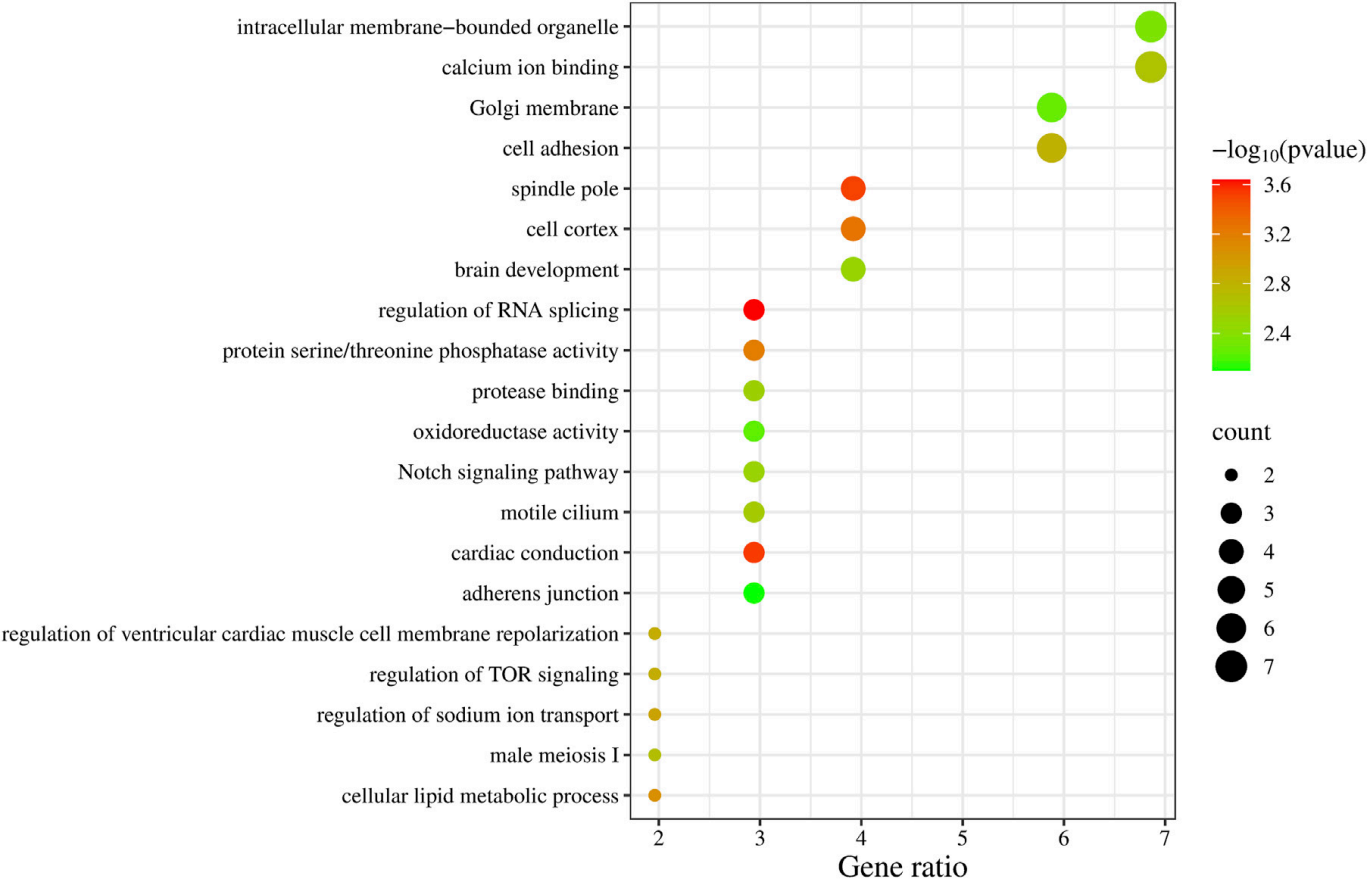
GO enrichment analysis results of 99 candidate genes.

We summarized the key biological functions of the positional candidate genes through GO enrichment (Figure 2), with calcium ion binding (GO:0005509) and intracellular membrane-bounded organelle (GO:0043231) having the highest number of candidate genes, each with 7 genes. The three most significant biological functions based on *P*-values (*P* < 5 × 10^−4^) include regulation of RNA splicing (*PTBP3*, *PRX*, *RBM38*), cardiac conduction (*SPTBN4*, *KCNH2*, *ZMPSTE24*), and spindle pole (*NEDD9*, *FRY*, *PLEKHG6*, *RAD21*).

**FIGURE 3:**
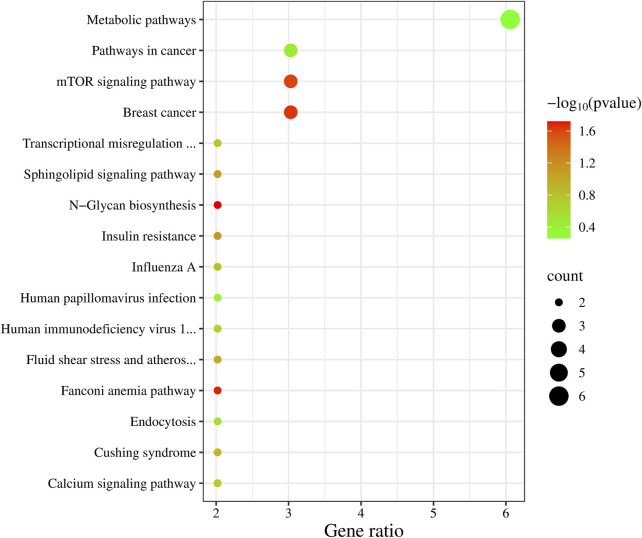
KEGG enrichment analysis results of 99 candidate genes.

In the KEGG enrichment analysis (Figure 3), the highest number of candidate genes, six in total (*NOS3*, *AGXT*, *MAN1C1*, *ITPKB*, *MGAT4C*, *BLVRB*), are involved in Metabolic pathways. Additionally, three candidate genes are associated with Breast cancer (*DLL3*, *BRCA2*, *WNT2B*), the mTOR signaling pathway (*WNT2B*, *TELO2*, *TNFRSF1A*), and Pathways in cancer (*DLL3*, *BRCA2*, *WNT2B*).

**FIGURE 4:**
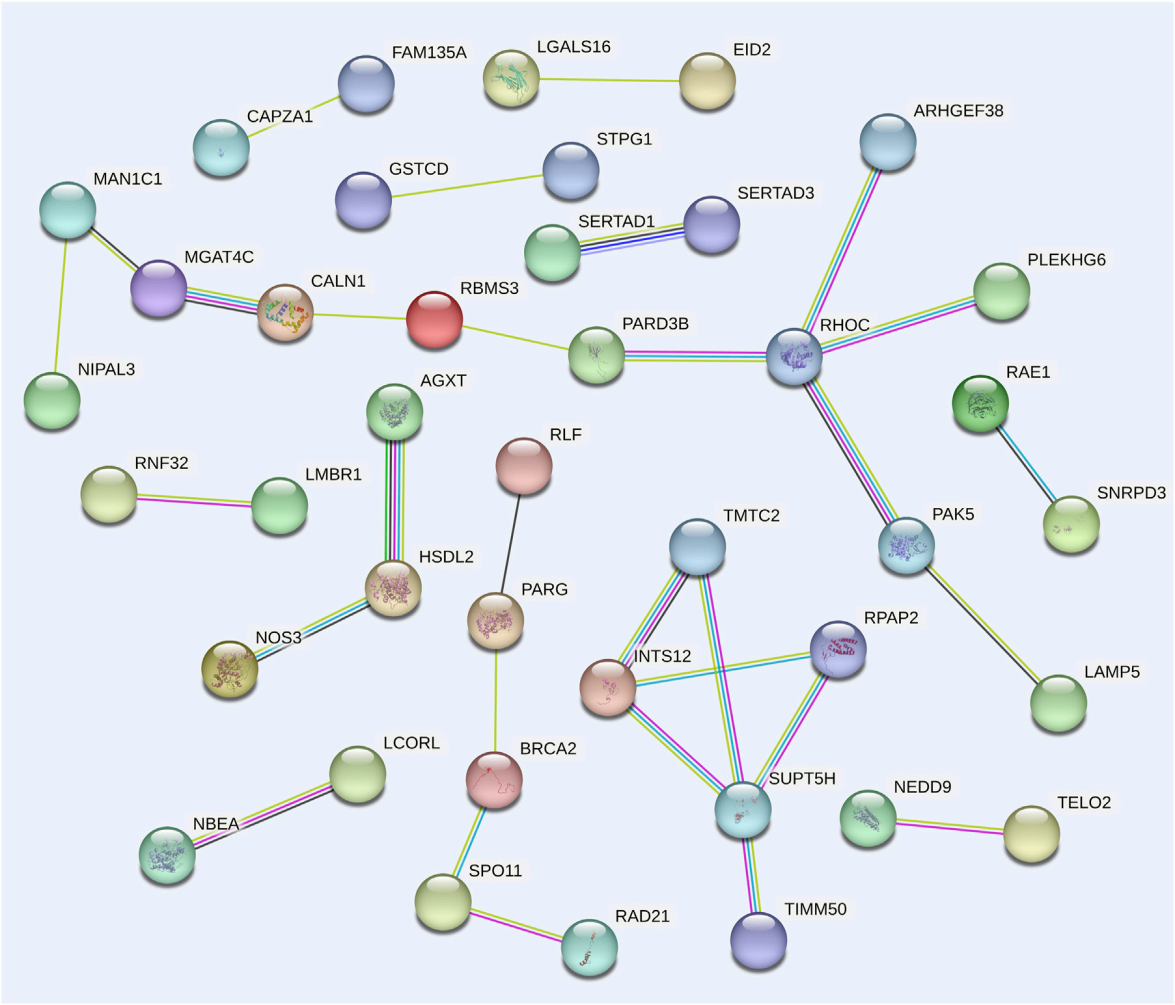
Gene interaction network composed of 32 candidate genes associated with various traits.

Using 99 candidate genes from all traits, a gene interaction network was constructed to visualize potential functional candidate genes (Figure 4). 41 candidate genes were predicted to have interactions, forming a total of twelve connected groups. The largest gene group consists of 11 genes. This gene network shows high connectivity (*P* = 2.34 × 10^−3^). Among these, *RHOC* and *SUPT5H* co-regulate four genes each, while *INTS12* interacts with three genes. These genes warrant prioritized investigation in subsequent functional studies.

## 4 Discussion

The milk production trait, being an essential characteristic in dairy goats, has been extensively studied in various breeds through high-density genotyping and GWAS to identify significant genetic markers and candidate genes related to economically important traits in goat milk. According to previous studies, genes such as *CSN1S1* (Massender et al., 2023), *CSN1S2* (Massender et al., 2023), *SOCS3* (Song et al., 2024), *SLC7A5* (Ni et al., 2024), *DGAT1* (Eid et al., 2020), and *INSIG1* (Wang et al., 2016) have all been associated with milk production traits in dairy goats. Among these, *CSN1S1*, *CSN1S2*, and *DGAT1* have been widely reported in studies on milk production traits in dairy goats. However, these genes were not linked to the traits in our results. We hypothesize that this is due to milk production being a complex quantitative trait influenced by both genetic and environmental factors, leading to varying association results across different dairy goat breeds. There may be genetic differences between dairy goat breeds, and the genomic structure, metabolic mechanisms, and development of the mammary glands in different breeds may have varying effects on milk production (Massender et al., 2023). The choice of statistical models and data analysis methods can also have a significant impact on the research results.

In GWAS analysis, the choice of model is crucial for the accuracy of the results. This study employed three models—GLM, MLM, and FarmCPU—to conduct GWAS analysis on milk production traits in dairy goats. Utilizing these three models is beneficial as different models may have varying sensitivities to different types of gene effects, such as additive effects, dominant effects, and epistatic effects. By using multiple models, the probability of detecting single nucleotide polymorphisms (SNPs) associated with the traits can be increased. Each model has different assumptions and algorithms for processing data. Employing various models helps to reduce bias and errors that may arise from the failure of assumptions in a single model, thus making the results more robust. The combined use of these models can fully leverage their respective strengths, thereby enhancing the overall effectiveness of the analysis (Visscher et al., 2017).

**FIGURE 5:**
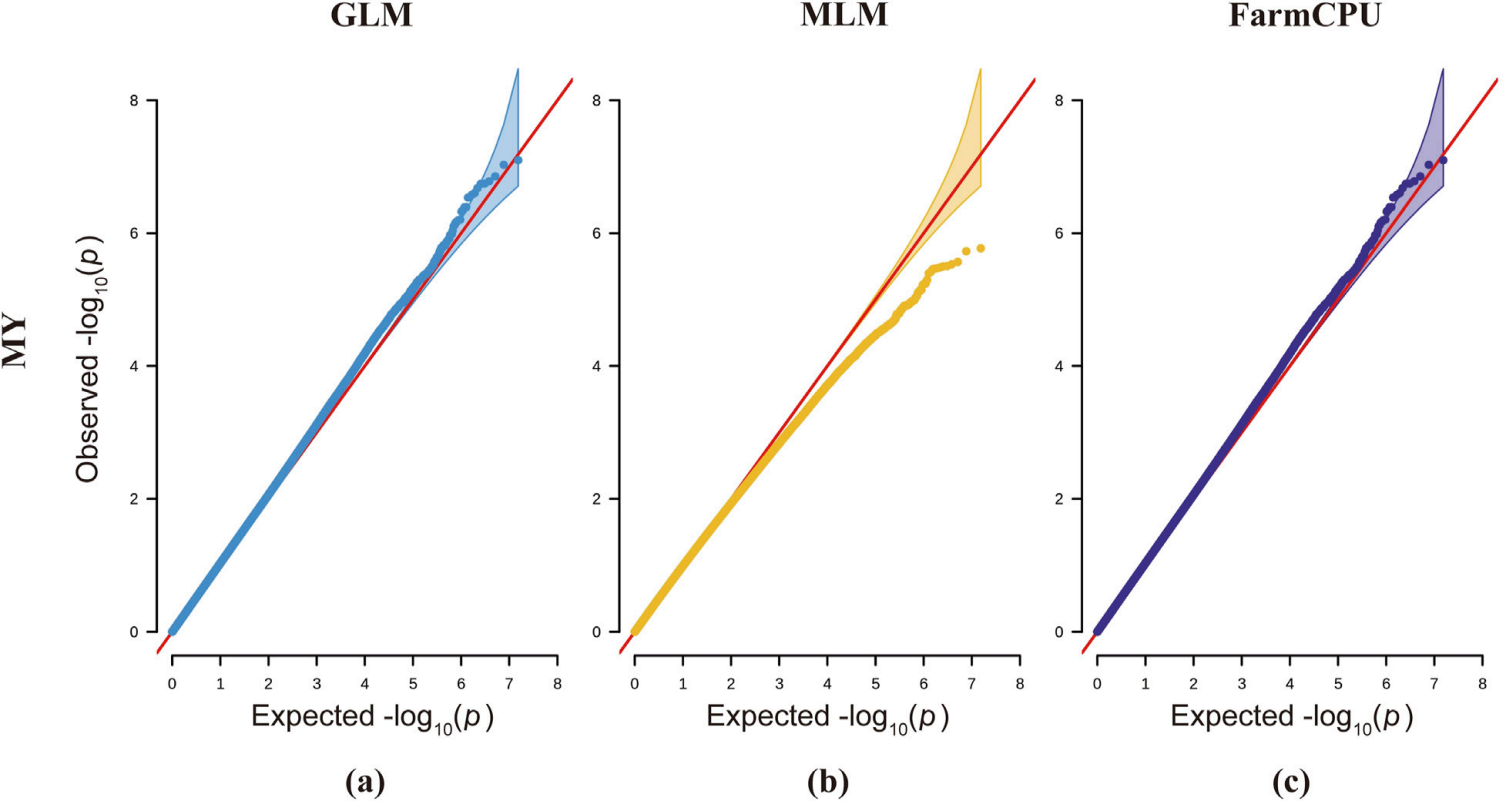
QQ plots for three different models across milk yield traits in dairy goats. The first column represents the GLM model (blue), the second column represents the MLM model (yellow), and the third column represents the FarmCPU model (purple). The red line represents the distribution of expected values, while the blue, yellow, and purple lines represent the actual distribution of observed values.

Each of the three analytical models presents distinct advantages and limitations. While the GLM detects a greater number of statistically significant SNP loci, it demonstrates an elevated probability of false positives (Price et al., 2006). The MLM, incorporating a kinship matrix, provides superior control over false positive results, though this comes at the cost of increased risk of false negatives (Yu et al., 2006). The FarmCPU model, with its built-in covariate correction capability, enables more comprehensive identification of significant SNP loci and candidate genes associated with complex traits; however, its stringent data requirements may lead to reduced detection of true positive loci (Liu et al., 2016). Integrating GLM, MLM, and FarmCPU models significantly expands the discovery of novel functional SNPs and enhances the detection of true positive loci, thereby identifying more candidate genes for downstream screening. However, this multi-model approach concomitantly increases false positive risks, necessitating rigorous experimental validation in subsequent studies. According to previous studies, the Bonferroni method for determining the threshold is highly restrictive, which may lead to the exclusion of some useful loci and result in false-negative outcomes (Armstrong, 2014). The QQ plots for milk yield trait analysis across all three models demonstrate systematic deviations of observed values from the expected probability distributions, indicating elevated risks of false negatives (Figure 5). Therefore, based on the actual situation, we adjusted the P-value threshold for milk yield traits to 5x10^−9^ for genome-wide signification and 1x10^−7^ for suggestive associations.

The lack of variability in lactose and total solids may be due to the biological characteristics of Guanzhong Dairy Goat, warranting further investigation. The range of variation in milk yield, milk fat percentage, milk protein percentage, and ash percentage suggests that these traits follow a normal distribution in the study samples. The high variability in somatic cell count may be attributed to the presence of mastitis in some dairy goats, leading to a significant increase in the number of somatic cells. In the milk of dairy goats tested in this group, the maximum somatic cell count exceeded 10000k/mL, while the minimum was 32k/mL, indicating significant differences between individuals (Tvarožková et al., 2023). However, it is known that the normal level of somatic cell count in dairy goats is below 1000k/mL, with excessive counts indicating the presence of mastitis (Smistad et al., 2025).

The genetic distances between all samples in the phylogenetic tree ranged from 0.1 to 0.3, indicating high genetic similarity among them (Supplementary Figure S1). This confirms that all samples belong to the same species. Principal component analysis (PCA) further supported this conclusion, as the samples clustered closely together (Supplementary Figure S2). However, slight dispersion of some samples suggests individual genetic variation within the population. Population structure analysis revealed that the optimal number of subgroups (K) was 2, reflecting genetic divergence among individuals (Supplementary Figures S3,S4). This may be attributed to the hybrid origin of Guanzhong dairy goats, which resulted from crossbreeding between Saanen goats and indigenous Chinese goats. The individual genetic differences observed in both PCA and population structure analyses could lead to population stratification, a major challenge in GWAS. Moreover, population stratification may introduce spurious associations, potentially generating numerous false-positive results in GWAS analyses (Haldar and Ghosh, 2012). Appropriate application of statistical models can effectively correct for population stratification and reduce false-positive associations (Sharma et al., 2015). Including principal components (PCs) from PCA as covariates in association analysis can effectively mitigate the impact of population stratification. The QQ plot showed minimal deviation of observed p-values from the expected distribution (λ = 1.06), while the genomic inflation factor (λ) remained within the acceptable threshold (0.94 < λ < 1.05) (Nazar et al., 2022). This approach can significantly reduce false-positive associations caused by population stratification.

For the milk yield trait (Figure 4), the QQ plot of the model indicates that the observed values for the MLM model deviate downward from the expected probability compared to the GLM and FarmCPU models, thereby increasing false negatives in the MLM model. In the QQ plot for the lactose trait, the p-values in the MLM model also show a slight downward deviation, indicating the presence of false negatives. In the QQ plot for somatic cell count using the GLM model, the p-values tilt upward prematurely at the front end, increasing the probability of SNP false positives (Supplementary Figure S5). The QQ plots for the FarmCPU model in traits such as ash content, somatic cell count, fat, and total solids show a line close to 1:1 with a sharp deviation at the tail (Supplementary Figure S5). In the QQ plot for the ash content trait in the FarmCPU model, the p-values exhibit the highest deviation, indicating the presence of SNP highly associated with this trait (Supplementary Figure S5).

We believe that the 18 candidate genes (*CCDC154*, *CLCN7*, *GNPTG*, *PTX4*, *TELO2*, *UNKL*, *UQCC4*, *BLVRB*, *HIPK4*, *PRX*, *SERTAD1*, *SERTAD3*, *SPTBN4*, *LCORL*, *SELENOF, RHOC, SUPT5H, INTS12*) identified in multiple traits are of significant importance for further research. Liao et al. (2012) found that upregulating *CCDC154* in HEK293 cells can inhibit cell proliferation by inducing G2/M arrest. Hong et al. (2023) found that *CCDC154* can promote the proliferation and metastasis of colorectal cancer cells. *CLCN7* was previously identified as a positional candidate gene for acute anterior uveitis (Huang et al., 2020). *CLCN7* expression is upregulated by the pain- and inflammation-associated mediator bradykinin (BK), thereby promoting osteoclast differentiation (Srivastava et al., 2014). *GNPTG* exerts a downregulatory effect on subclinical endometritis in dairy cows, effectively suppressing inflammatory responses (Hoelker et al., 2012). The pentraxin family includes *PTX1* and *PTX2* (*NPTX1* and *NPTX2*, respectively), *PTX3*, and *PTX4* (Li et al., 2023). These genes serve as a link between immunity and inflammation, and as a bridge between innate and adaptive immunity (Li et al., 2023). However, *PTX4* is less studied and requires more research to elucidate its function in immunity (Li et al., 2023). The *PRX* gene has been identified to be strongly associated with sheep wool follicle development and wool shedding, playing an important role in promoting follicle formation, epidermal differentiation, and follicle stem cell development (Lei et al., 2021). Extracellular *PRX* family proteins released from necrotic brain cells activate Toll-like receptor 2 (*TLR2*) and *TLR4* in macrophages, upregulating inflammatory cytokines such as *IL-23* and subsequently promoting neural cell death (Ito et al., 2013). Using *SERTAD1* knockout mice and various molecular methods revealed that *SERTAD1* can regulate NLRP3-mediated inflammasome activation (Ha et al., 2024). Studies have reported the inhibitory effect of *SERTAD1* on apoptosis/anoikis (Nguyen et al., 2022). In the GWAS analysis of milk production traits in Thai dairy cattle, *LCORL* was identified as a positional candidate gene for milk total solids percentage (Buaban et al., 2022). *LCORL* gene-associated quantitative trait loci (QTL) are related to the somatic cell score in Valle del Belice Dairy Sheep (Mohammadi et al., 2022). *SELENOF* can affect the proliferation and death of normal epithelial and breast cancer cells by regulating p21 and p27 (Ekyalongo et al., 2023). Lipopolysaccharide (LPS) induces inflammatory responses in cells while upregulating SELENOF expression (Lee et al., 2022). According to research results and related gene studies, most genes are associated with cancer, inflammation, and cell proliferation. Mastitis, as a common inflammation, also leads to cell proliferation and may phenotypically affect milk composition traits (Paixão et al., 2017). Therefore, the genes *CCDC154*, *CLCN7*, *GNPTG*, *PTX4*, *PRX*, *SERTAD1, RHOC, SUPT5H, INTS12* and *SELENOF* may potentially be associated with somatic cell count (SCC) and mastitis susceptibility. Although the genes *TELO2*, *UNKL*, *BLVRB*, *HIPK4*, *SERTAD3*, *SPTBN4*, and *UQCC4* demonstrated significant associations with multiple traits, no published studies have yet established their relationship with milk production traits, warranting further experimental validation. Among them, the *LCORL* gene is related to milk production traits, and further research may help to explore its potential function in dairy goat milk production traits.

Among the 99 positional candidate genes identified, we found 5 genes associated with milk production traits through literature review: *TNFRSF1A*, *SPATA6*, *MAN1C1*, *MASP1*, and *BRCA2*. In cattle, higher mRNA expression levels of *TNFRSF1A* correlate with lower proliferation rates of mammary cells but higher apoptosis rates (Orellana Rivas et al., 2021). Lopreiato et al. (2020) identified high transcript abundance of *TNFRSF1A* through MIXED model variance analysis, indicating its important role in immune response and inflammation. *SPATA6* was identified as a positional candidate gene for somatic cell score traits in Romanian dairy cows through GWAS (Ilie et al., 2021). *MAN1C1* was determined as a target candidate gene for bovine milk oligosaccharides (Poulsen et al., 2019) and lactation persistence (Do et al., 2017) according to GWAS. A polymorphism in *MASP1*, SNP g.5766A>G, is associated with milk protein percentage (Zhang et al., 2019). *BRCA2* increases the risk of breast cancer in women and promotes the proliferation and differentiation of mammary epithelial cells (Vidarsson et al., 2002). Mammary epithelial cells secrete milk and synthesize its major components, and their proliferation and differentiation impact milk production traits.

## 5 Conclusion

This study identified SNPs and positional and functional candidate genes significantly associated with milk production traits in dairy goats using three GWAS models: GLM, MLM, and FarmCPU. The GWAS analysis results showed a total of 242 significant SNPs corresponding to 99 positional candidate genes within a 50 kb upstream and downstream range. 15 significant SNP loci were consistently identified across all three models, corresponding to 18 candidate genes. The integrated analysis of three models detected 2, 19, 17, 4, 115, 23, and 62 significant SNPs associated with milk yield, ash percentage, protein percentage, lactose percentage, somatic cell count, fat percentage, and total dry matter percentage, respectively. Correspondingly, 6, 24, 9, 12, 37, 14, and 30 candidate genes were identified for these traits. Additionally, we proposed several new candidate genes related to milk production traits (*LCORL*, *TNFRSF1A*, *SPATA6*, *MAN1C1*, *MASP1*, *BRCA2*). In summary, this study provides an important basis for subsequent research.

## Data Availability

The datasets presented in this study can be found in online repositories. The names of the repository/repositories and accession number(s) can be found below: https://ngdc.cncb.ac.cn/gsa, CRA031559.

## Supplementary material

SUPPLEMENTARY FIGURE S1 Phylogenetic tree of 208 dairy goats.

SUPPLEMENTARY FIGURE S2 Population structure demonstrated by principal component analysis.

SUPPLEMENTARY FIGURE S3 Cross-validation error values at different K values.

SUPPLEMENTARY FIGURE S4 Population structure plot of 208 dairy goats. The value of K ranges from 2 to 5, in descending order.

SUPPLEMENTARY FIGURE S5 QQ plots for three different models across six traits in dairy goats. The first column represents the GLM model (blue), the second column represents the MLM model (yellow), and the third column represents the FarmCPU model (purple). AP = Ash Percentage; PP = Protein Percentage; LP = Lactose Percentage; SCC = Somatic Cell Count; FP = Fat Percentage; TDM = Total Dry Matter Percentage. The red line represents the distribution of expected values, while the blue, yellow, and purple lines represent the actual distribution of observed values.

SUPPLEMENTARY FIGURE S6 Manhattan plots for six traits in dairy goats across three different models. **(a)** — AP; **(b)** — PP; **(c)** — LP; **(d)** — SCC; **(e)** — FP; **(f)** — TDM. Each plot, from top to bottom, represents the GLM, MLM, and FarmCPU models. On the Y-axis, the red line indicates the significance level of p ≤ 0.00001.
