# Supplementary figures and images for: Genome-wide association study on dairy goat milk production traits using three models
Source: Front Genet. 2025 Aug 22;16:1650836. doi: 10.3389/fgene.2025.1650836 (PMC12411178; doi:10.3389/fgene.2025.1650836)

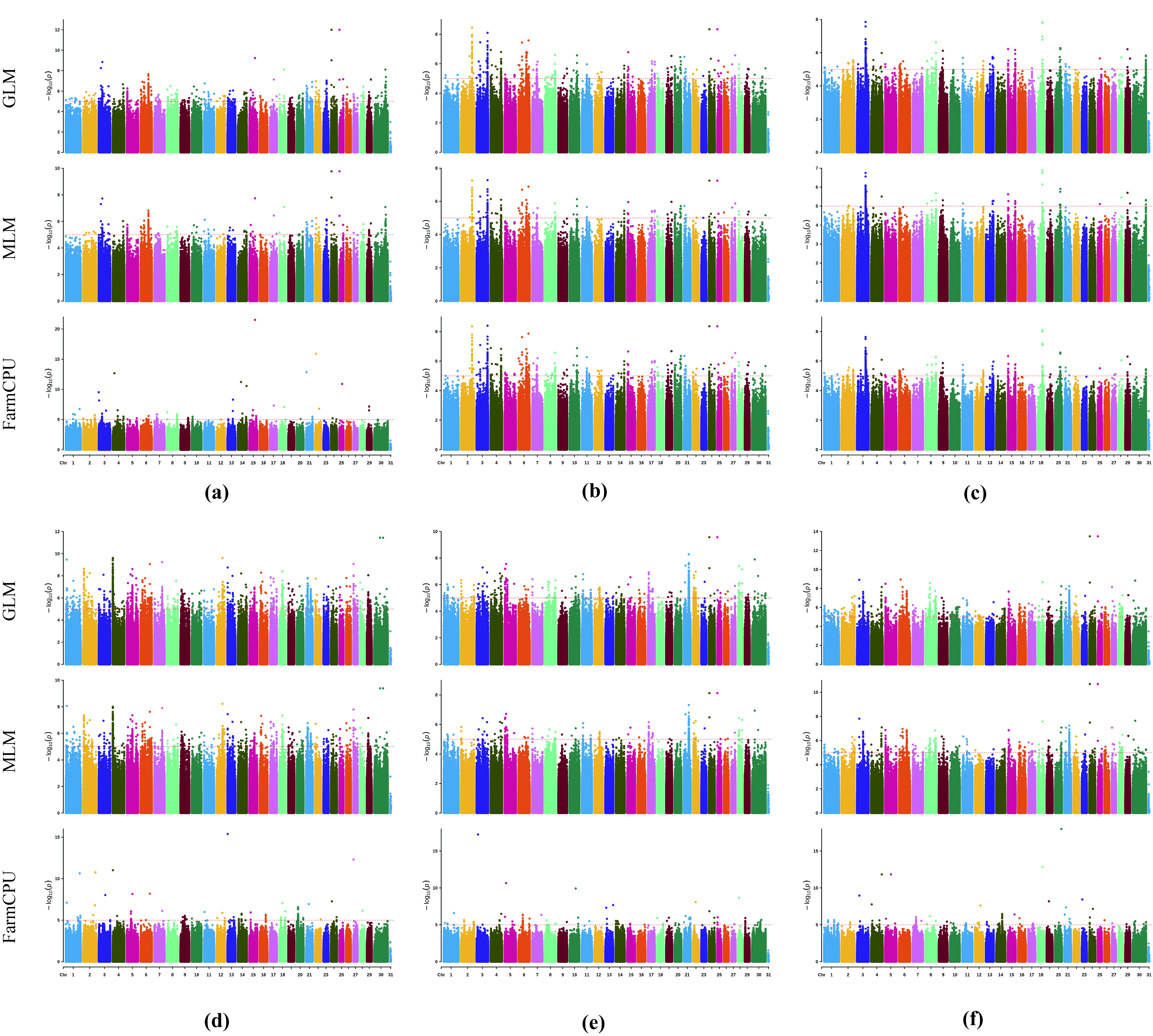

Supplement: Supplementary file 1 [file Image6.tif]

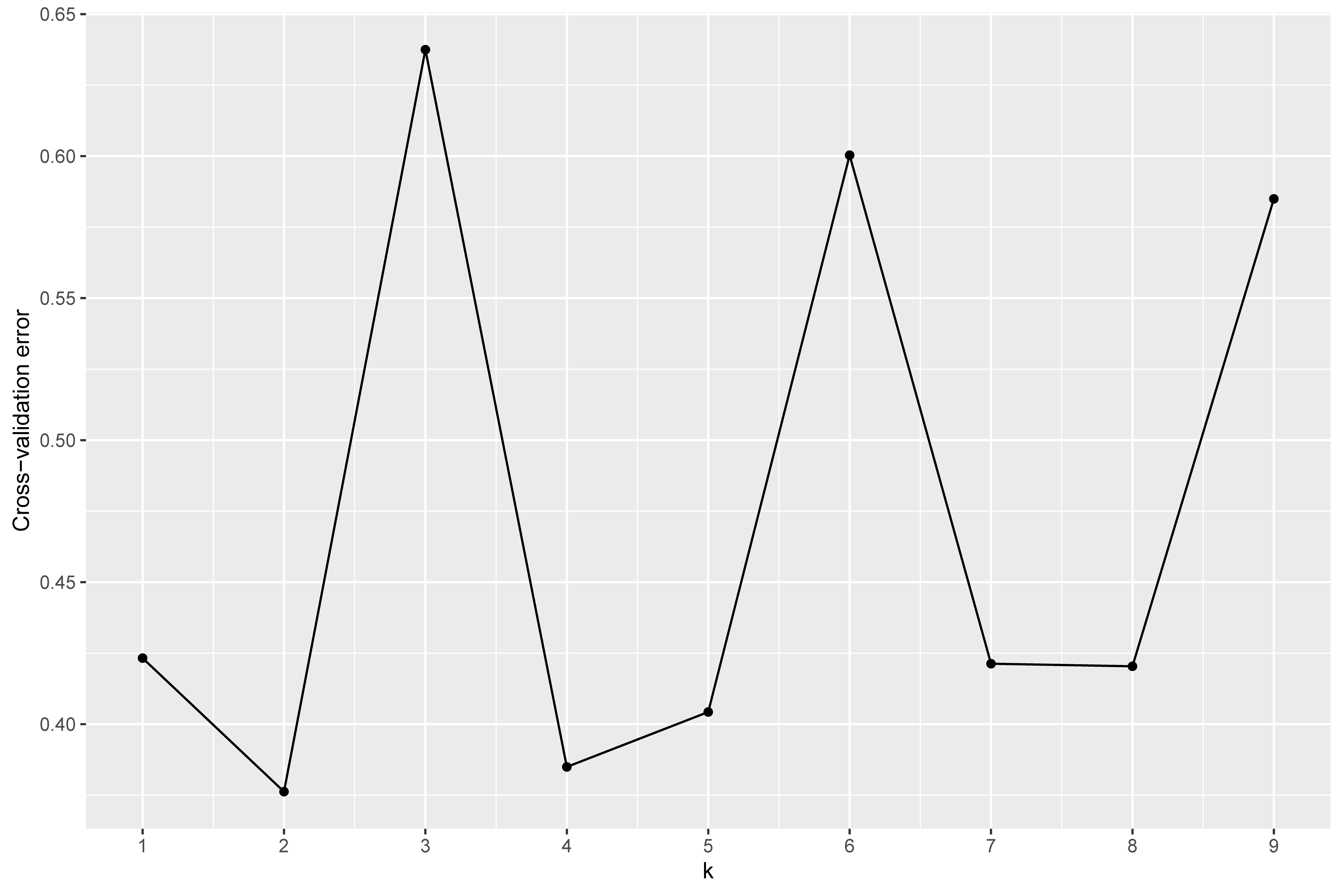

Supplement: Supplementary file 3 [file Image3.tif]

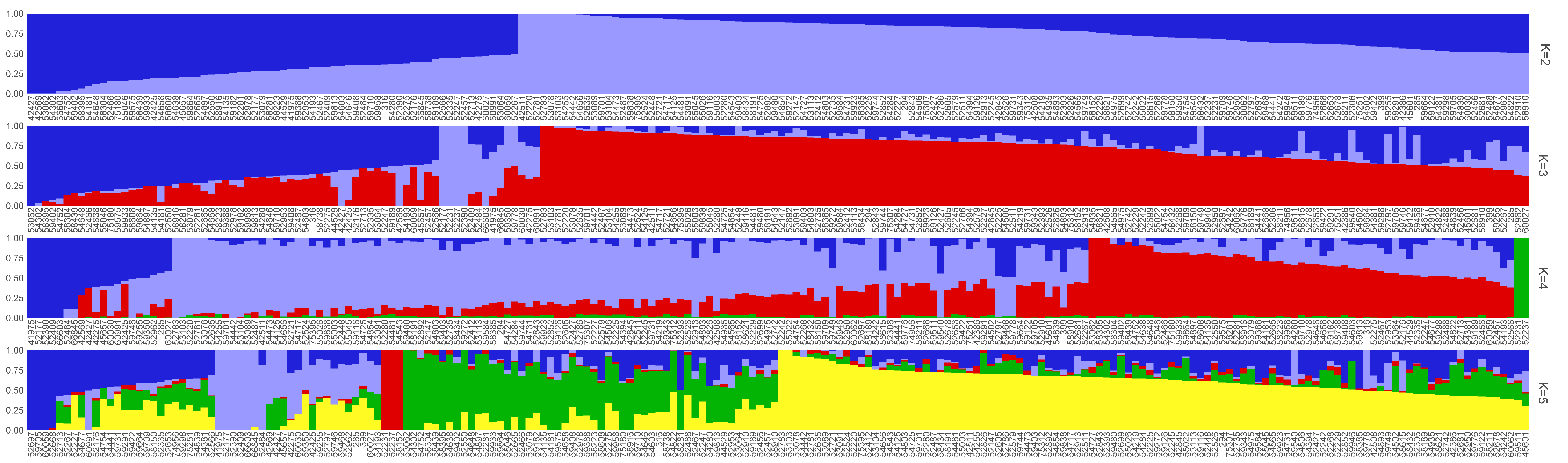

Supplement: Supplementary file 4 [file Image4.tif]

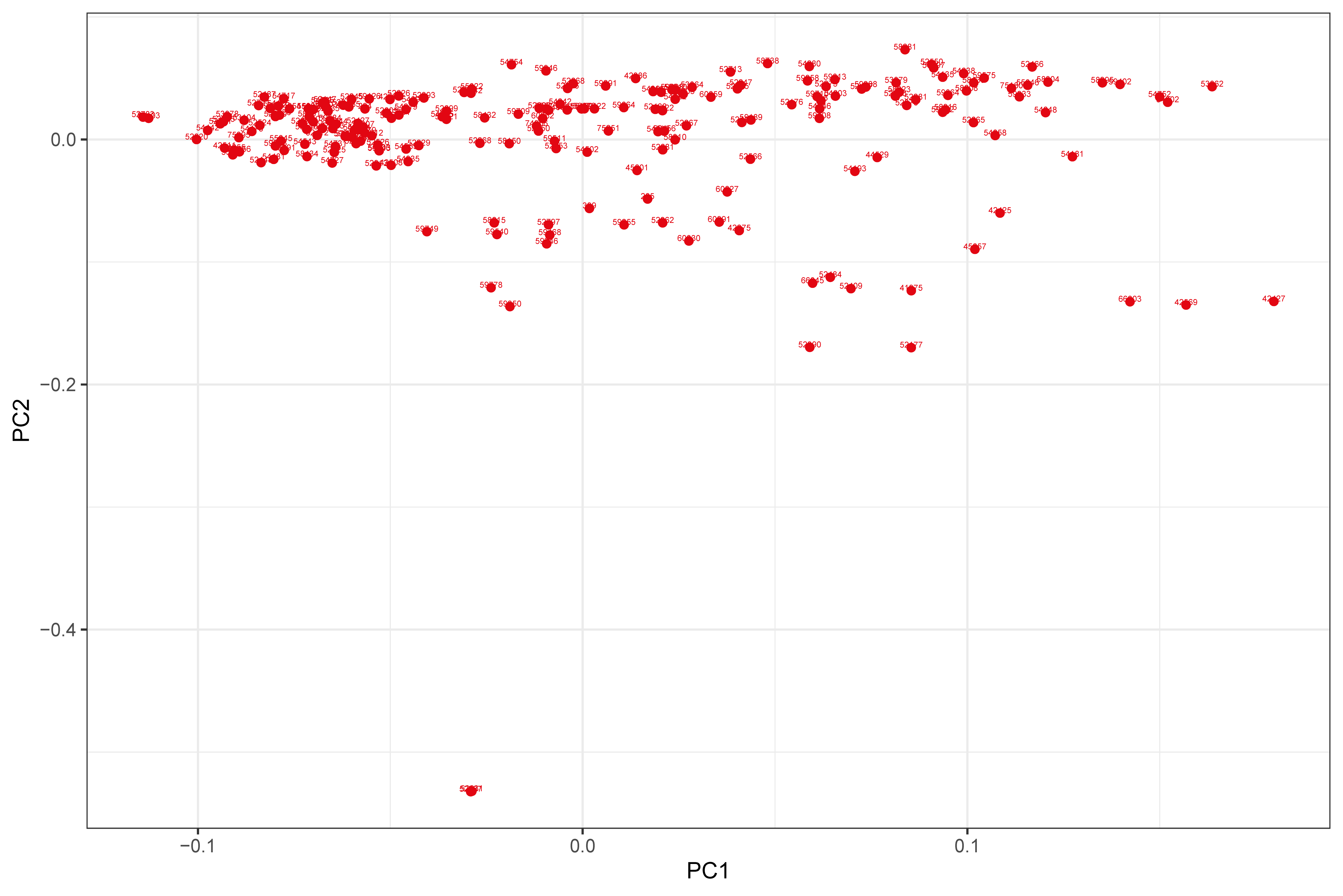

Supplement: Supplementary file 5 [file Image2.tif]

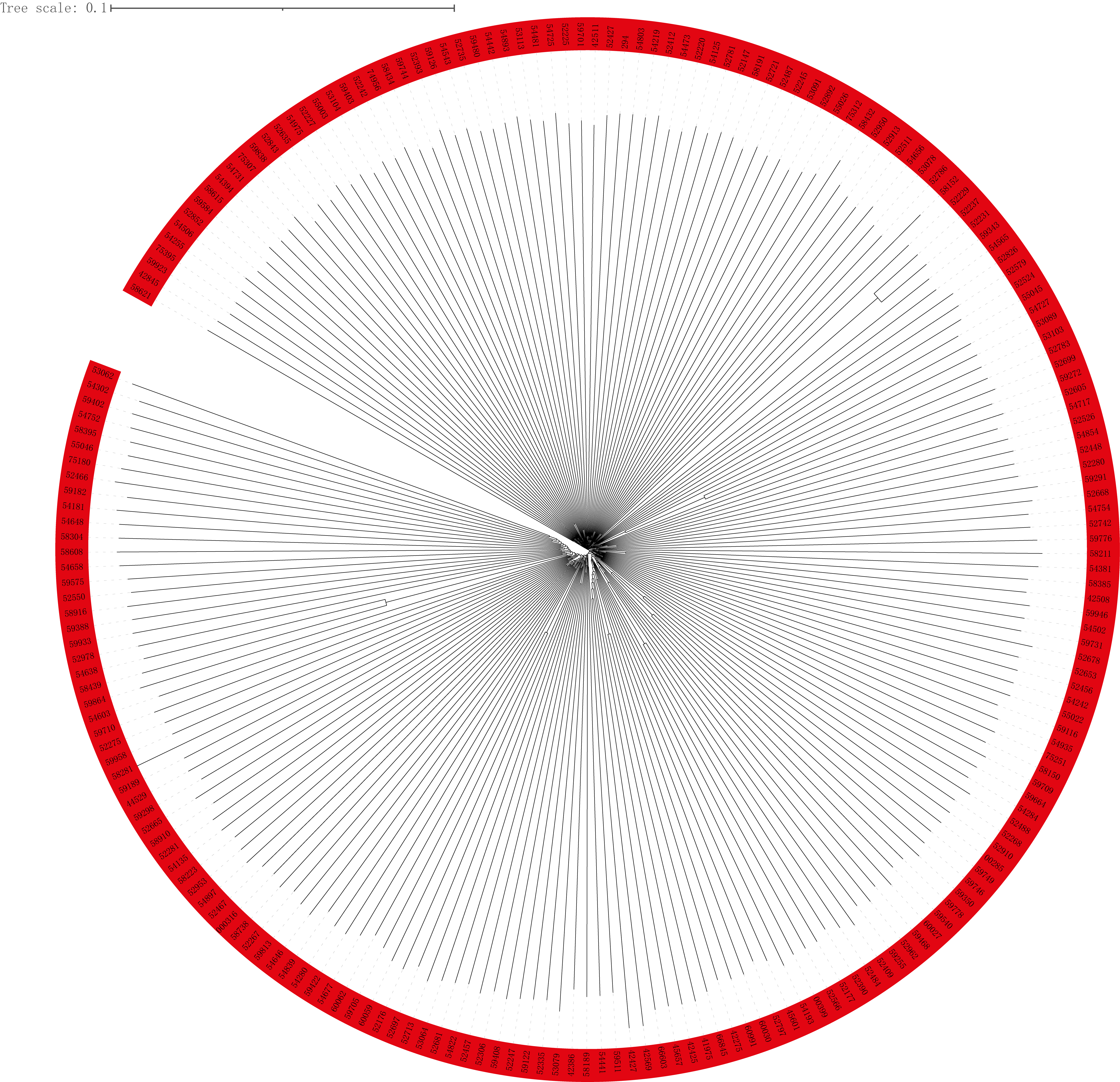

Supplement: Supplementary file 6 [file Image1.tif]
