# Supplementary material for: Genome-wide association study on dairy goat milk production traits using three models
Source: Front Genet. 2025 Aug 22;16:1650836. doi: 10.3389/fgene.2025.1650836 (PMC12411178; doi:10.3389/fgene.2025.1650836)
Supplement: Supplementary file 2 [file Table1.docx]

Table S1. Chromosome- and genome-wise significant SNP and positional candidate genes related to each trait identified by three different models

|  |  |  | **-Log_10_P^3^** | | |
| --- | --- | --- | --- | --- | --- |
| **Trait^1^** | **Position^2^** | **Gene name** | **GLM** | **MLM** | **FarmCPU** |
| MY | 5:102961538 | *SCNN1A,TNFRSF1A,PLEKHG6* | 7.10 | — | 7.10 |
| FP | 25:1040722 | *PTX4,GNPTG,TELO2,CCDC154,CLCN7,UQCC4,UNKL* | 9.57 | 8.12 | — |
|  | 5:4662526 | *CAPS2* | 7.20 | — | — |
|  | 3:14938807 | *RLF,ZMPSTE24,TMCO2* | — | — | 17.24 |
|  | 5:15642325 | *MGAT4C* | 7.56 | — | 10.66 |
|  | 3:64111333 | *SELENOF,HS2ST1* | 7.29 | — | — |
| PP | 25:1040722 | *PTX4,GNPTG,TELO2,CCDC154,CLCN7,UQCC4,UNKL* | 8.33 | 7.25 | 8.36 |
|  | 6:38045361 | *LCORL* | 7.44 | — | 7.62 |
|  | 3:115965324 | *PBX1* | 7.25 | — | 7.46 |
|  | 3:115965481 | *PBX1* | 8.10 | 7.28 | 8.40 |
| LP | 18:50080860 | *TIMM50,EID2,DLL3,SUPT5H,LGALS16* | 7.84 | — | 8.11 |
|  | 18:50080885 | *TIMM50,EID2,DLL3,SUPT5H,LGALS16* | 7.80 | — | 8.00 |
|  | 3:89856257 | *7SK,WNT2B,ST7L* | 7.85 | — | 7.50 |
|  | 3:89978023 | *PPM1J,MOV10,CAPZA1,RHOC* | 7.58 | — | 7.63 |
| AP | 3:909995 | *AGXT,MAB21L4* | — | — | 9.53 |
|  | 25:1040722 | *PTX4,GNPTG,TELO2,CCDC154,CLCN7,UQCC4,UNKL* | 12.00 | 9.76 | — |
|  | 3:4841299 | *IQCA1* | — | — | 8.17 |
|  | 3:23014256 | *SPATA6* | 8.25 | 7.30 | — |
|  | 25:28482819 | *CALN1* | 10.90 | — | — |
|  | 14:33261840 | *TRPS1* | — | — | 11.21 |
|  | 18:50682477 | *SERTAD3,SERTAD1,SPTBN4,HIPK4,BLVRB,PRX* | 8.11 | — | — |
|  | 13:58115430 | *RAE1,RBM38,SPO11* | — | — | 8.30 |
|  | 14:91327385 | *FAM135A,SDHAF4* | — | — | 10.56 |
| TDM | 25:1040722 | *PTX4,GNPTG,TELO2,CCDC154,CLCN7,UQCC4,UNKL* | 13.49 | 10.68 | — |
|  | 23:5385191 | *NEDD9* | — | — | 8.43 |
|  | 4:6375779 | *NOS3,KCNH2* | — | — | 7.77 |
|  | 6:38045361 | *LCORL* | 7.29 | — | — |
|  | 6:38078839 | *LCORL* | 7.64 | — | — |
|  | 8:45629567 | *PTAR1,APBA1* | 7.90 | — | — |
|  | 8:45629967 | *PTAR1,APBA1* | 7.42 | — | — |
|  | 8:46657959 | *TRPM3* | 7.35 | — | — |
|  | 8:46769905 | *TRPM3* | 7.38 | — | — |
|  | 18:50682477 | *SERTAD3,SERTAD1,SPTBN4,HIPK4,BLVRB,PRX* | 8.66 | 7.57 | 12.85 |
|  | 12:57987611 | *ZAR1L,FRY,BRCA2* | — | — | 7.61 |
|  | 20:59387521 | *DNAH5* | 7.34 | — | — |
|  | 3:64111333 | *SELENOF,HS2ST1* | 7.42 | — | — |
|  | 3:64195062 | *HS2ST1* | 7.64 | — | — |
|  | 8:101430783 | *HSDL2,PTBP3* | 7.74 | — | — |
|  | 4:108098809 | *PON1* | 7.24 | — | — |
|  | 4:108101143 | *PON1* | 7.60 | — | — |
|  | 4:112336848 | *CDK14* | 8.15 | — | 11.84 |
| SCC | 4:125152 | *VIPR2* | 7.46 | — | — |
|  | 4:138927 | *VIPR2* | 7.80 | — | — |
|  | 4:146422 | *VIPR2* | 7.95 | — | — |
|  | 4:172076 | *VIPR2* | 8.31 | — | — |
|  | 4:239363 | *VIPR2* | 9.00 | 7.48 | — |
|  | 4:261523 | *VIPR2* | 7.40 | — | — |
|  | 4:1310530 | *UBE3C* | 8.17 | — | — |
|  | 4:1314545 | *UBE3C* | 7.44 | — | — |
|  | 4:1328157 | *UBE3C* | 9.61 | 7.91 | — |
|  | 4:1340006 | *UBE3C* | 7.91 | — | — |
|  | 4:1342390 | *UBE3C* | 8.76 | 7.21 | — |
|  | 27:1370511 | *ZNF385D* | 8.02 | — | — |
|  | 27:1375152 | *ZNF385D* | 7.98 | — | — |
|  | 27:1388247 | *ZNF385D* | 9.07 | 7.80 | 12.31 |
|  | 27:1391960 | *ZNF385D* | 7.26 | — | — |
|  | 4:1400114 | *UBE3C* | 8.80 | 7.32 | — |
|  | 4:1647280 | *LMBR1* | 7.25 | — | — |
|  | 4:1656942 | *LMBR1* | 7.34 | — | — |
|  | 4:1658113 | *LMBR1* | 7.29 | — | — |
|  | 4:1670723 | *LMBR1* | 7.43 | — | — |
|  | 4:1670732 | *LMBR1* | 8.59 | 7.39 | — |
|  | 4:1670750 | *LMBR1* | 8.17 | — | — |
|  | 4:1671158 | *LMBR1* | 9.54 | 8.00 | 11.02 |
|  | 4:1677198 | *LMBR1* | 7.25 | — | — |
|  | 4:1678808 | *LMBR1* | 7.70 | — | — |
|  | 4:1687974 | *LMBR1,RNF32* | 7.29 | — | — |
|  | 4:1688699 | *LMBR1,RNF32* | 7.41 | — | — |
|  | 4:1689491 | *LMBR1,RNF32* | 7.66 | — | — |
|  | 4:1690073 | *LMBR1,RNF32* | 7.25 | — | — |
|  | 28:1822142 | *PARG* | 7.38 | — | — |
|  | 13:2275920 | *LAMP5,PAK5* | 8.75 | 7.44 | 15.38 |
|  | 13:2277317 | *LAMP5,PAK5* | 7.34 | — | — |
|  | 22:4538859 | *RBMS3* | 7.74 | — | — |
|  | 2:7445261 | *STPG1,NIPAL3* | 8.61 | 7.32 | — |
|  | 2:7445386 | *STPG1,NIPAL3* | 8.64 | 7.35 | — |
|  | 2:7446903 | *STPG1,NIPAL3* | 8.05 | — | — |
|  | 2:7447079 | *STPG1,NIPAL3* | 8.37 | — | — |
|  | 2:7447171 | *STPG1,NIPAL3* | 8.25 | — | — |
|  | 2:7447208 | *STPG1,NIPAL3* | 7.76 | — | — |
|  | 2:7447919 | *STPG1,NIPAL3* | 8.18 | — | — |
|  | 2:7449903 | *STPG1,NIPAL3* | 8.27 | — | — |
|  | 2:7452767 | *STPG1,NIPAL3* | 7.57 | — | — |
|  | 2:7453232 | *STPG1,NIPAL3* | 8.33 | — | — |
|  | 2:7454359 | *STPG1,NIPAL3* | 7.20 | — | — |
|  | 17:7614488 | *FAM222A* | 7.83 | — | — |
|  | 17:7614633 | *FAM222A* | 7.86 | — | — |
|  | 2:8518161 | *LDLRAP1,MAN1C1* | 7.26 | — | — |
|  | 5:12323695 | *U6,TMTC2* | 7.34 | — | — |
|  | 16:14147064 | *BRINP3* | 8.28 | 7.32 | — |
|  | 21:16180128 | *AGBL1* | 7.29 | — | — |
|  | 21:16264900 | *AGBL1* | 7.81 | — | — |
|  | 21:16324371 | *AGBL1* | 7.77 | — | — |
|  | 21:16400098 | *AGBL1* | 7.30 | — | — |
|  | 6:19761195 | *ARHGEF38,GSTCD,INTS12* | 7.61 | — | — |
|  | 6:19882280 | *ARHGEF38* | 7.43 | — | — |
|  | 16:28177588 | *ITPKB,STUM* | 7.47 | — | — |
|  | 18:30898086 | *CDH8* | 7.23 | — | — |
|  | 18:30898955 | *CDH8* | 7.21 | — | — |
|  | 14:34682675 | *RAD21* | 8.21 | — | — |
|  | 2:42405457 | *PARD3B* | 7.88 | — | — |
|  | 3:50403121 | *TNNI3K* | 8.10 | — | — |
|  | 5:58558861 | *NEUROD4* | 8.00 | — | — |
|  | 12:60915290 | *NBEA* | 9.60 | 8.22 | — |
|  | 12:62950314 | *UFM1* | 7.43 | — | — |
|  | 12:62950396 | *UFM1* | 7.45 | — | — |
|  | 12:62950398 | *UFM1* | 7.45 | — | — |
|  | 12:62955177 | *UFM1* | 7.44 | — | — |
|  | 3:69436777 | *C1orf146,RPAP2,GLMN* | — | — | 8.03 |
|  | 1:79753572 | *RTP1,MASP1* | 7.55 | — | — |
|  | 7:83146505 | *GRAMD2B* | 9.23 | 7.89 | — |
|  | 1:146711134 | *U2* | — | — | 10.64 |

^1^MY = Milk Yield; AP = Ash Percentage; PP = Protein Percentage; LP = Lactose Percentage; SCC = Somatic Cell Count; FP = Fat Percentage; TDM = Total Dry Matter Percentage.

^2^Significant SNP sites are represented as chromosome: base pair.

^3^Dashs indicates that the SNP site corresponding to this model is not significant.
